# Eye movements powered by artificial intelligence identify asymptomatic carriers of familial Alzheimer’s disease

**DOI:** 10.1093/braincomms/fcaf370

**Published:** 2025-09-25

**Authors:** Gerardo Fernández, Luis Mendez, Francisco Lopera, David Aguillon, Mario A Parra

**Affiliations:** Viewmind Inc., New York, Alpine, NJ 07620, USA; Grupo de Neurociencias, Facultad de Medicina, Universidad de Antioquia, Medellín 050030, Colombia; Grupo de Neurociencias, Facultad de Medicina, Universidad de Antioquia, Medellín 050030, Colombia; Grupo de Neurociencias, Facultad de Medicina, Universidad de Antioquia, Medellín 050030, Colombia; Department of Psychological Sciences & Health, University of Strathclyde, Glasgow G1 1QE, UK

**Keywords:** familial Alzheimer’s disease, visual short-term memory, eye-tracking, artificial intelligence

## Abstract

Eye-tracking (ET) metrics obtained during the Visual Short-Term Memory Binding Task (VSTMBT) have shown promise in detecting early and subtle alterations in individuals at risk for, or diagnosed with, Alzheimer’s disease (AD) dementia. However, there remains a critical need for robust, automated classification methods capable of delivering affordable digital biomarker solutions for the preclinical detection of AD. This study aimed to address this need. A sample of 100 carriers (89 healthy asymptomatic carriers—HAC and 11 symptomatic familial Alzheimer’s disease—FAD) of the E280A mutation in PSEN1 from the widely investigated cohort in Antioquia, Colombia, and 119 healthy controls (Controls HCA: 91 and Controls FAD: 28) participated in the study. The groups were assessed using the novel VSTMBT coupled with ET and an extensive neuropsychological battery. Oculomotor behaviours were recorded using ET, and their analysis was based on Machine Learning classification using Random Forest (RF) Models. Classification accuracy incorporated both true and false positives and negatives. The RF models that incorporated oculomotor behaviours accurately identified FAD (Accuracy = 100%) and HAC (Accuracy = 96%), outperforming classification accuracy based on pure behavioural scores (FAD = 98% and HAC = 73%). The cognitive biomarker drawn from RF models that incorporated oculomotor behaviours accurately detected mutation carriers who inevitably develop FAD and outperformed traditional forms of cognitive assessment. The oculomotor phenotype unveiled here characterizes the preclinical stages of FAD, as it has been identified in most carriers, even those in the still asymptomatic stages.

## Teaser

Fernández *et al*. report that eye-tracking metrics during the Visual Short-Term Memory Binding Task, analysed with Random Forest models, accurately identified asymptomatic and symptomatic carriers of E280A-PSEN1 familial Alzheimer’s disease. The findings support oculomotor behaviour as a sensitive digital biomarker for early, preclinical detection of Alzheimer’s disease.

## Graphical Abstract

**Graphical Abstract:**
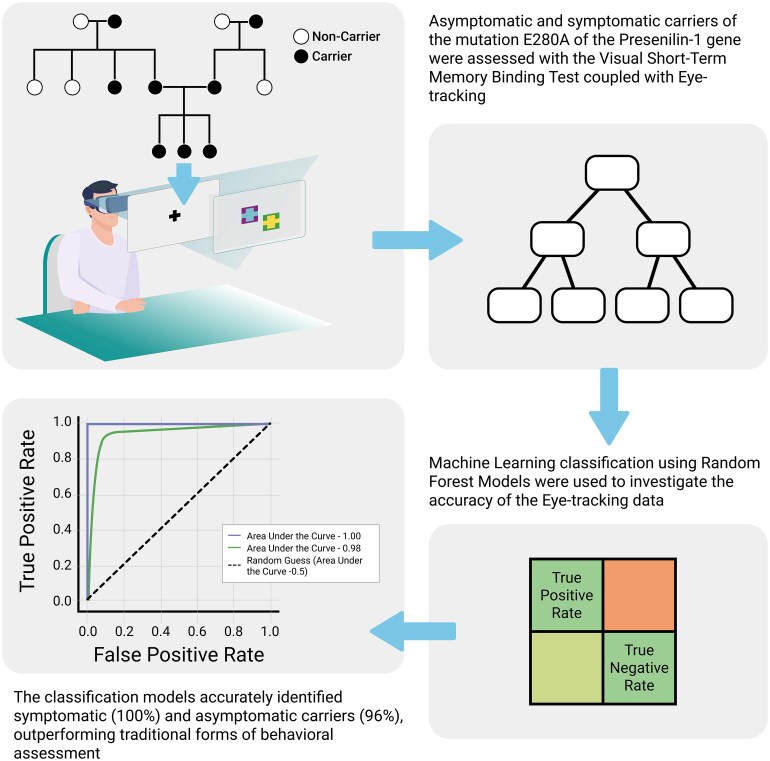

## Introduction

Available neuropsychological screening tests are frequently informative about advanced stages of neurodegenerative diseases but fail to detect their earlier stages (i.e. limited sensitivity) and the underlying disorder (i.e. lack of specificity).^1,2^ Moreover, such tests can be influenced by the individual’s socio-cultural background.^3,4^ Advanced neuroimaging methods, adhering to the new biomarker framework,^5^ have good predictive value in the early stages of such diseases. However, their costs are prohibitive, and their availability is limited,^6^ especially for underrepresented populations. Blood-based biomarkers are proving to be promising in the preclinical detection of Alzheimer’s disease (AD).^7^ Yet, their introduction in research and clinical settings still faces challenges. For instance, the EU/US CTAD Task Force^7^ envisions that blood-based biomarkers will be used in primary care settings to identify patients who will then require referral for further cognitive evaluation by specialists in memory disorders. This highlights an existing gap in the healthcare pathway due to the lack of reliable and easy-to-use cognitive screening tests. The availability of affordable, scalable and complementary biomarkers (i.e. peripheral blood-based and neurocognitive biomarkers) in clinical and research settings will represent a significant step forward in the era of dementia prevention and brain health promotion.^8^

Regarding peripheral neurocognitive biomarkers, recent evidence suggests that the eye can be a window to unveil the functional, neuropathological and cognitive impacts of early neurodegeneration in preclinical AD.^9,10^ Recent findings indicate that oculomotor behaviours associated with cognitive performance can reveal neurocognitive characteristics of Alzheimer’s disease (AD), thereby improving the accuracy of its assessment. By integrating eye-tracking (ET) with the evaluation of Visual Short-Term Memory Binding (VSTMB)—a cognitive function recognized as a preclinical marker of AD^11^—we have shown that fixation patterns and pupillary responses can reliably differentiate individuals with suspected AD dementia from cognitively healthy older adults.^2^

Furthermore, by subjecting such data to prospective prediction algorithms, the risk of progressing to AD or non-AD dementia among patients with Mild Cognitive Impairment (MCI) was detected with 94% and 100% accuracy, respectively.^2^ These recent findings support the proposal that oculomotor behaviours linked to cognitive performance on tests that are sensitive and specific to AD, such as the VSTMB Task (VSTMBT), can provide reliable neurocognitive biomarkers for screening dementia risk.

The VSTMBT assesses the ability to temporarily hold visual arrays of multi-feature objects as individual (i.e. only colours or shapes) or unified (e.g. bicoloured objects^11^ or coloured shapes^12^) representations (i.e. study arrays) and compares these with subsequent arrays (i.e. test arrays), which show the same objects/features or different objects (i.e. features recombined in the test array) or features (i.e. features not shown in study array). Two versions of the task have been previously used to assess carriers of the E280A-PSEN1 mutation, who inevitably develop familial Alzheimer’s disease (FAD).^11,12^ The tasks were administered when the participants were either cognitively unimpaired, as determined by traditional neuropsychological tests, or in the early stages of FAD. Using the shape-colour task,^11^ the authors reported a sensitivity of 73% and 77% for each group, respectively. For the colour-colour version of the task (used in this study), the authors reported a sensitivity of 76% and 77% for each group, respectively. The VSTMBT has been recommended as a promising cognitive test for the preclinical assessment of AD.^1^ Recent studies have confirmed that performance on the shape-colour test correlates with AD biomarkers in members of this kindred,^13^ in patients with sporadic MCI,^14^ and in cognitively unimpaired older adults.^15^ The task has accurately differentiated between patients with Subjective Cognitive Decline (SCD) and healthy controls,^16^ and has proved accurate in predicting conversion from MCI to AD dementia, as shown by behavioural^17^ and previous ET^2^ studies (the latter using the colour-colour version). The fact that both versions of the VSTMBT have achieved similar outcomes suggests a genuine impairment of binding functions in VSTM in AD, which seems to be independent of the task used and the disease variant (i.e. sporadic or familial).

By combining the assessment of VSTMB with the simultaneous recording of oculomotor behaviours, we have observed that the sensitivity of the assessment of patients with AD dementia can be enhanced (i.e. relative to just behavioural scores).^18^ It has recently been suggested that by relying on Artificial Intelligence (AI) methodologies, the role of ocular biomarkers for early detection of AD can be unveiled and significantly enhanced.^9^ We hypothesized that the novel neurocognitive biomarker (i.e. VSTMB and ET) powered by AI would boost the sensitivity of the assessment and reveal impairments in asymptomatic carriers of the E280A-PSEN1 who are not identified by traditional neuropsychological tests or novel cognitive markers.^11,12^ It is worth noting that the presence of this mutation leads to FAD in 100% of carriers who first develop MCI around the age of 44 years and dementia in their 50s.^19^

The literature reporting on this rare form of AD (i.e. E280A-PSEN1) has confirmed that its cognitive phenotype is indistinguishable from that seen in the more prevalent variants of sporadic AD.^20,21^ This, together with the understanding that this mutation has 100% penetrance, creates an unparalleled opportunity to identify the earliest cognitive and clinical manifestations of AD. Giudicessi *et al*.^20^ acknowledged that insights drawn from autosomal dominant AD, such as that caused by the E280A-PSEN1 mutation, can inform prevention initiatives targeting sporadic AD variants. Using the VSTMBT incorporated in the present study, Parra *et al*.^11^ reported that deficits in VSTMB were indistinguishable between familial and sporadic AD. In their sample of asymptomatic carriers of the E280A-PSEN1 mutation, such deficits were the only observable manifestation of the disease. Recent studies have confirmed that VSTMB deficits observed in cognitively unimpaired or mildly impaired older adults are associated with the accumulation of amyloid-β.^14,15^ There are no reliable genetic tests to detect the risk of dementia due to sporadic AD. Therefore, affordable biomarker solutions that rely on valid cognitive assessments are highly desired. This study aims to contribute to this outstanding need.

## Materials and methods

### Participants

The sample consisted of 89 asymptomatic carriers who have a respective control group (*n* = 91) and 11 symptomatic carriers who also have a respective control group (*n* = 28).

**Figure 1:**
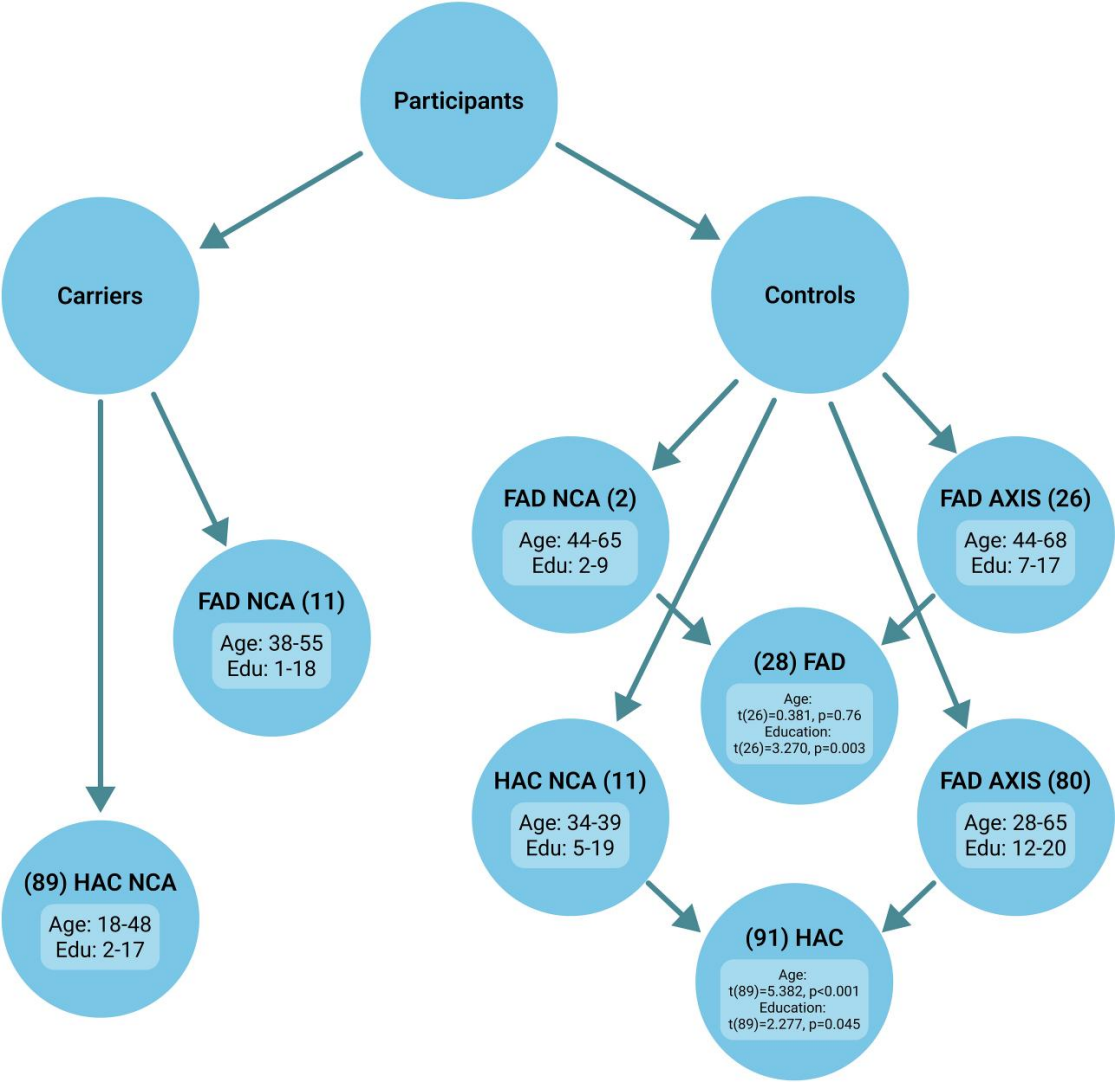
**Diagram illustrating the recruitment procedures towards the sample design (FAD, familial Alzheimer’s disease; HAC, healthy asymptomatic carriers; NCA, Neuroscience Centre Antioquia, Colombia; AXIS, AXIS Neurociencias, Bahía Blanca, Buenos Aires, Argentina.** Age and Education ranges are presented for each group. *T*-tests were carried out between controls from different centres. All the mutation carriers (HAC and FAD) and 13 Controls (FAD NCA and HAC NCA) were prospectively recruited at the Health Unit of the Neuroscience Centre of the University of Antioquia (NCA). To increase power for RF analysis, we drew a retrospective sample of healthy controls (for FAD cases, FAD AXIS, and asymptomatic carriers, HAC AXIS) who had already been assessed with the same oculomotor biomarker at AXIS Neurociencias using a different ET device (see Supplementary Table 2 for more information on the data drawn from these devices).

Mutation carriers were members of a large kindred from the Colombian province of Antioquia, South America. They carry the gene mutation E280A of PSEN1, which inevitably leads to an autosomal-dominant early-onset FAD. Individuals carrying mutations, whether in the symptomatic or asymptomatic stages of the disease, routinely participate in clinical and research evaluations at the Health Unit of the Neuroscience Centre, University of Antioquia. The genetic status of these participants remains blinded to the clinical and research staff throughout the study. Upon completion of the recruitment process, genetic information was provided in an anonymized format using coded identifiers, ensuring participant confidentiality. The control sample consisted of healthy older adults prospectively recruited at the Health Unit of the Neuroscience Centre of the University of Antioquia (*n* = 13) and retrospectively selected from the AXIS Neurociencias (Bahía Blanca, Buenos Aires, Argentina) (*n* = 106). The latter were allocated to the control groups for FAD and healthy asymptomatic carriers (HAC), keeping the range of age and education as close as possible to that of the controls recruited at the Neuroscience Centre of the University of Antioquia (see Fig. 1 for sample design). The study protocols were approved by the Ethics Committee at the University of Antioquia, Colombia and the Ethics Committee of the Hospital Municipal de Agudos (Bahía Blanca, Buenos Aires, Argentina).

Carriers were classified into FAD or HAC according to the criteria proposed by Acosta-Baena *et al*.^19^ To apply such criteria, we relied on the neuropsychological protocol used by the Neuroscience Group of Antioquia, which has recently published its updated norms.^22^ We also used these norms to identify patients’ domain-specific impairments (see Supplementary Table 1).

Healthy participants (all non-carriers) entered the control groups if they had (i) a negative history of neurological or psychiatric disorders, (ii) a Mini-Mental State Examination (MMSE) score > 24 and (iii) no memory complaints as documented by a self-report and family questionnaire. This cut-off value for the MMSE can be considered a low threshold. However, a recent clinical trial involving asymptomatic carriers of the autosomal-dominant mutation who did not meet criteria for MCI or AD dementia^23^ used an MMSE cut-off of 24 for participants with <9 years of education. More recently, to investigate the impact of social determinants of health on cognition, researchers^24^ divided a Colombian sample into low (fewer than 23 points in the MMSE) and high cognitive functioning (above 24). As Fig. 1 illustrates, some controls recruited from Colombia had a very low level of education. This, together with the blind nature of our recruitment process, led us to adopt a lower MMSE cut-off for normality. Each participant underwent a colour vision assessment using Dvorine pseudo-isochromatic plates.^25^

### Neuropsychological assessment

We relied on the Neuropsychological test battery of the Neuroscience Centre of Antioquia, which has been used for more than two decades to assess individuals with or at risk of AD.^22,26,27^ Some of these tests are part of the CERAD (i.e. CERAD-Colombia^26,27^) and have been validated in Colombia.^28^ For example, the Word List Test of the CERAD-Colombia has identified the earliest cognitive impairment in HAC at an average age of 32 years, which is 12 and 17 years before the median ages of MCI and dementia onset, respectively.^29^ The battery was comprised of the Spanish translations of the MMSE,^30^ the PAL Task,^31^ Verbal (Letter-FAS)^32^ and Animal Fluency Tests,^33^ the Copy and Recall of the Complex Figure of Rey-Osterrieth,^34^ Part A of the Trail Making Test,^35^ the Boston Naming Test,^36^ the Wisconsin Card Sorting Test^37^ and the Word List Test.^27^ We also included a functional scale^38^ and Subjective Cognitive Scales (participants and family members) in the local protocol.^39^

Only controls from the Neuroscience Centre of Antioquia underwent a full neuropsychological assessment, as the recruitment process in our study was conducted in a blind manner. The controls, retrospectively recruited from AXIS Neuroscience (see Fig. 1), were relatives of patients who agreed to participate in other studies. Only an interview and a cognitive screening tool (MMSE) were administered to ascertain their cognitively unimpaired status. The MMSE scores for all the participants from AXIS Neuroscience were within the normal range (mean: 29.65, SD: 2.25, median: 29 and range: 27–30).

### The VSTMBT and ET

**Figure 2:**
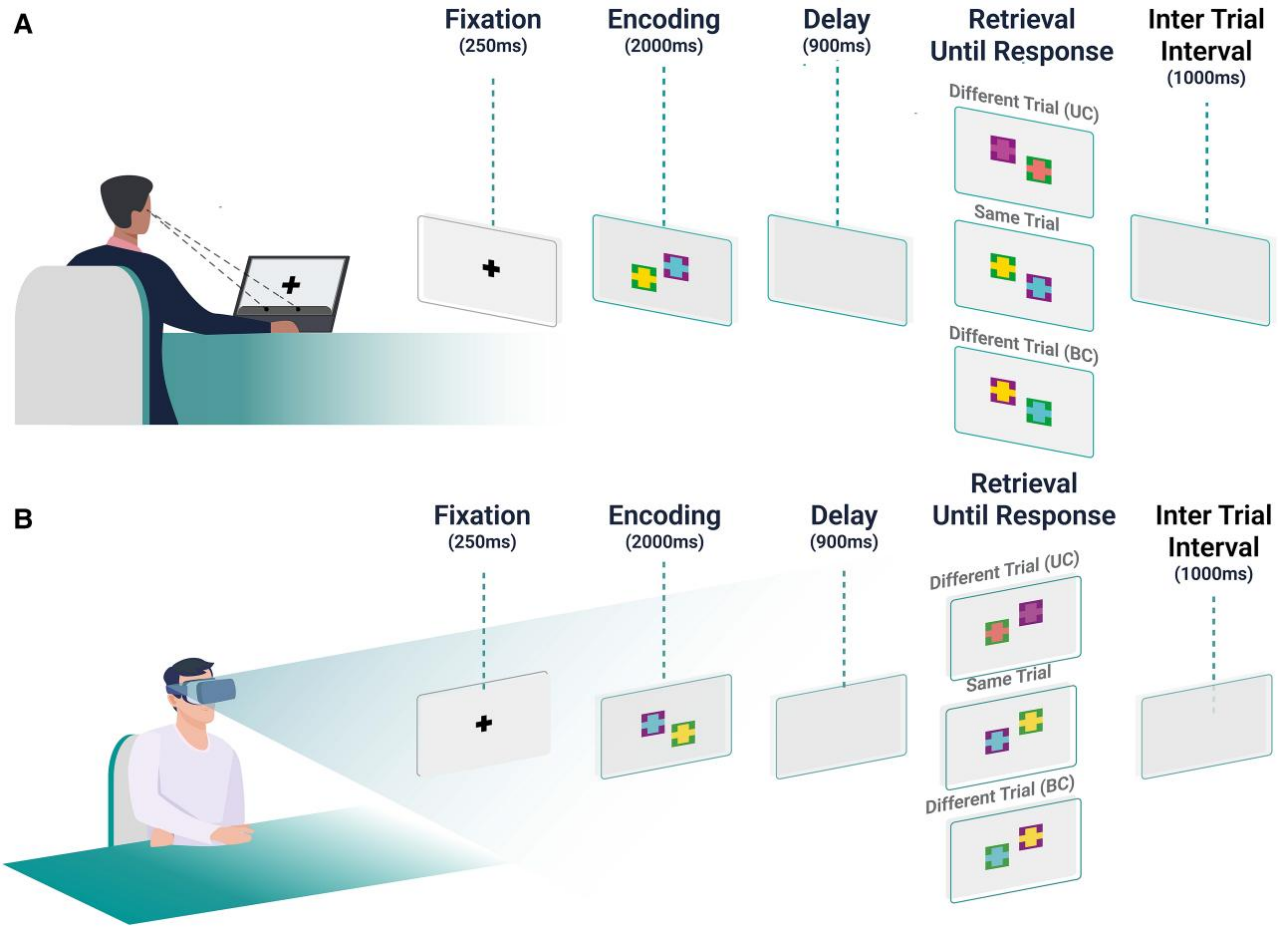
**Testing setting and an example of a trial of the VSTMBT.** (**A**) A desktop eye-tracker was a GazePoint (https://www.gazept.com/medical/). (**B**) The HMD (HP Reverb Omniset, Hewlett-Packard, Palo Alto, CA, USA, with Eye Tracking).

The VSTMBT is a change detection paradigm that assesses the ability to temporarily hold bicoloured objects whose colours have to be retained as individual features (unbound colours—UC) or integrated within the objects (bound colours—BC). During the task, participants detect changes happening between a study and a test display separated by a 1 s retention interval. Changes could be in the form of new colours replacing colours previously studied (UC) or colours swapping between bi-coloured objects (BC). The study and test display consisted of arrays of two items each for the FAD patients and their controls and of three items for HAC and their controls. This approach aimed to replicate the methodology used in the behavioural study by Parra *et al*.^11^ We were interested in investigating whether, under such stimulation conditions, oculomotor behaviours could provide additional insights. The dependent variable was the Percentage of Correct Recognition. We have previously identified the oculomotor metrics that are sensitive to alterations compatible with cognitive impairment in subjects with AD dementia. We have reported that gazing,^40^ number of fixations,^40^ fixation duration,^40^ and pupil behaviour^2^ are promising variables for that aim. We collected ET data with two different devices: a desktop eye-tracker and a head-mounted eye-tracker (see Fig. 2A). In the desktop eye-tracker, visual stimuli were presented on the centerline of a 19″ LCD Monitor (1024 × 768 pixels resolution). Participants sat at a distance of 60 cm from the monitor. Head movements were minimized using a chin rest. Eye movements (i.e. fixation duration and saccade amplitude) were recorded with a GazePoint eye tracker, with a sampling rate of 150 Hz (https://www.gazept.com/medical/). The Head-Mounted Display (HMD) (HP Reverb Omniset, Hewlett-Packard, Palo Alto, CA, USA, with Eye Tracking) had the ViewMind software running on a computer suitable for the mentioned equipment (https://vr.tobii.com/integrations/hp-reverb-g2-omnicept-edition/). In Supplementary Table 2, we present the results from *t*-tests comparing the data (dependent variables: Fixation Duration, Number of Fixations, Pupil and Gazing) across the two ET devices (independent variable). All stimuli were generated using ViewMind’s registered algorithms (https://www.viewmind.com). Stimuli were presented on the centre line of the HMD. Eye movements were recorded with a sampling rate of 120 Hz. All recordings and calibrations were binocular. The stimulus was constructed following the layouts developed by Parra *et al*.^11^ (Fig. 2B). We performed ANOVA analyses to verify that the oculomotor measures gathered by both devices are compatible and comparable; none of the variables used as income of the RF showed significant differences when comparing the metrics from both devices.

### Data analysis

#### Group comparisons

Demographic data were compared using one-way ANOVA with Tukey’s honestly significant differences corrected *post hoc* tests (Table 1). Neuropsychological data from FAD and HAC were compared against the local norms.^27^ To explore VSTMB impairments in carriers of the mutation E280A,^11^ we ran a univariate general linear model (GLM) with Group (Controls FAD versus FAD versus Controls HAC versus HAC) as the between-subjects factor, years of education and age as covariates and the Cost of Binding as the dependent variable. The Cost of Binding is the cost of processing BC relative to processing individual colours (UC).^41^ We applied the formula provided by Parra *et al*. (2024)^15^ to our paradigm (Cost of Binding = (Performance UC − Performance BC)/Performance UC × 100). We derived measures of effect size (partial eta squared, *η*^2^: small = 0.01, medium = 0.06 and large = 0.14) and power (*β*) from the GLM.

**Table 1:** Demographic and neuropsychological variables for the two groups of carriers

	Controls for FAD (*n* = 28)	FAD (*n* = 11)	*P*-value or^a^ below norms	Controls for HAC (*n* = 91)	HCA (*n* = 89)	*P*-value or^a^ below norms
	Mean	Mean		Mean	Mean	
Years of Education	11.68 (3.23)	7.64 (6.30)	*P* = 0.017	15.27 (2.93)	10.43 (3.65)	*P* < 0.001
Age	56.11 (7.60)	47.00 (5.50)	*P* < 0.001	41.58 (8.44)	32.66 (8.17)	*P* < 0.001
Gender (F/M)—*Χ*^2^	15/13	4/7	*P* = 0.333	46/45	59/30	*P* = 0.025
MMSE	29.71 (0.81)	24.36 (3.64)	*P* < 0.001	29.62 (0.68)	28.78 (1.53)	*P* < 0.001
World List Learning (Total) (CERAD)		9.55 (3.98)	8		19.82 (4.11)	0
ROF Copy		19.15 (8.52)	5		31.57 (4.24)	0
ROF Recall		4.40 (2.80)	5		19.12 (7.11)	0
TMTA (time)		137.36 (100.02)	3		46.27 (20.95)	0
FAS		28.36 (14.78)	1		32.79 (8.64)	0
Category Fluency (CERAD)		14.18 (4.94)	2		19.97 (4.20)	0
WCST Categories		1.55 (1.21)	3		3.45 (1.55)	0
WCST Perseverations		17.36 (7.50)	2		13.69 (5.83)	0
WAISIII Digit to Symbol		21.80 (15.74)	5		55.92 (18.23)	0
Raven Matrices		6.91 (2.30)	1		9.92 (1.42)	0
IADL		5.80 (2.39)	5		8.00 (0.00)	0
QMF		24.09 (13.35)	4		5.89 (5.47)	0
QMP		20.73 (10.33)	4		10.94 (5.90)	0

Norms: controls were only screened for normal cognitive abilities using the MMSE and assessed with the novel neurocognitive biomarker. Available norms were used to verify the clinical and cognitive status of the assessed patients (Torres *et al*.,^22^ see Table 1 and Supplementary Table 1). *Χ*^2^, Chi-square performed.

^a^We applied the criteria proposed by Acosta-Baena *et al*.,^19^ which considered −2SD from the norm as the cut-off for impairment. The HACs who were classified as Asymptomatic pre-MCI were due to their MMSE scores. No domain-specific cognitive function was below 2SD from the norm. The between-group differences in the MMSE remained after controlling for the differences in Age and Education.

Although previous reports have confirmed that VSTMB is a function insensitive to demographic [i.e. education and age,^42^ socioeconomic^43^ and IQ variables (i.e. verbal IQ)],^15^ we explored such notions in this new dataset. Demographic variables (i.e. age and years of education) were first entered as covariates in the GLM. We then ran binomial correlations to explore, in the sample of mutation carriers, whether demographic (i.e. years of education) and fluid IQ variables (i.e. Raven Matrices) correlate with the Cost of Binding and the UC and BC scores.

#### AI classification for eye-tracking data

Our AI framework relied on Random Forest (RF) models. RF is a supervised machine learning algorithm based on ensemble learning, which constructs a multitude of decision trees during training and outputs the class that is the mode of the individual trees’ predictions. It operates by aggregating predictions across trees trained on bootstrapped subsets of the data, with random feature selection at each split, thereby reducing overfitting and improving generalization performance. In the context of eye movement analysis, RF models are advantageous due to their ability to model complex, non-linear relationships among a large number of oculomotor features (i.e. fixation duration, number of fixations, pupil and gazing). These features often exhibit intricate interactions that traditional statistical models struggle to capture. Moreover, RF models are robust to noise, do not require strong distributional assumptions and provide measures of feature importance, making them highly suitable for distinguishing between clinical populations, such as individuals with AD dementia, MCI and controls.

Two main RF models were defined to explore the classification power of ET data collected during the BC condition of the VSTMBT. Barral *et al*.^44^ reported that RF classification using ET data outperformed demographic and vocal biomarkers (AUC: 0.73 ± 0.04, 0.62 ± 0.03 and 0.71 ± 0.02, respectively) in discriminating between patients with AD, MCI and SCD. The first RF model identified HAC versus Controls. The second model identified FAD versus Controls (see Fig. 1 for the composition of each group). For each RF model, we conducted a stepwise approach wherein we combined eye movement variables. A 5-fold cross-validation approach over nine combinations of hyperparameter values was used to assess the RF model. Our model consisted of various decision trees, each slightly different from the others (see Supplementary Tables 3 and 4 for the Out-Of-Fold performance of all trained models). Using the majority voting algorithm,^45^ the ensemble makes predictions based on each individual decision tree model (bagging). As a result, the amount of overfitting is reduced while maintaining the classification ability. To evaluate the performance of the RF when classifying subjects, the following measures were defined. (i) True Positive Rate (TPR) measures indicate the proportion of actual positives which are correctly identified. (ii) True Negative Rate (TNR) measures indicate the proportion of actual negatives which are correctly identified. (iii) Positive and Negative Predictive Values (PPV and NPV) indicate the proportion of the true positive/negative against all the positive/negative results (both true positives and false positives). The overall accuracy indicates the proportion of true results (both true positives and true negatives) in the population (See Supplementary Fig. 1 for the formulas used to obtain these classification measures).

## Results

The neuropsychological and demographic data are presented in Table 1. Relative to their corresponding controls, FAD patients showed poorer general cognitive abilities measured by the MMSE. The HAC also showed a group effect on the MMSE. This was explained by four HACs who scored 2 SD below the norms on this test (see Supplementary Table 1). These were classified as Asymptomatic pre-MCI following the recommended criteria. While impairments in traditional neuropsychological tests were often found in FAD patients when compared to their local norms (i.e. >2 SD below the norm), no HAC exhibited performance that fit such a criterion (see Table 1 and Supplementary Tables 1 and 5).

### VSTMB performance

The GLM revealed a significant effect of Group [*F*(3,215) = 4.99, *P* < 0.001, *η*^2^ = 0.105, *β* = 0.98]. Bonferroni corrected *post hoc* tests confirmed Controls FAD versus FAD were significantly different (mean difference = 10.48, *P* = 0.019 and CI = −19.83/−1.125). Controls HAC versus HAC were also significantly different (mean difference = 5.25, *P* = 0.003 and CI = −9.17/−1.33). Neither the Control groups (mean difference = 0.94, *P* = 1.000 and CI = −6.63/4.73) nor the Carrier Groups (mean difference = 4.28, *P* = 1.000 and CI = −4.12/12.68) significantly differed (see Supplementary Fig. 2). We, therefore, decided to subject the BC Score to receiver operating characteristic (ROC) analysis.

**Figure 3:**
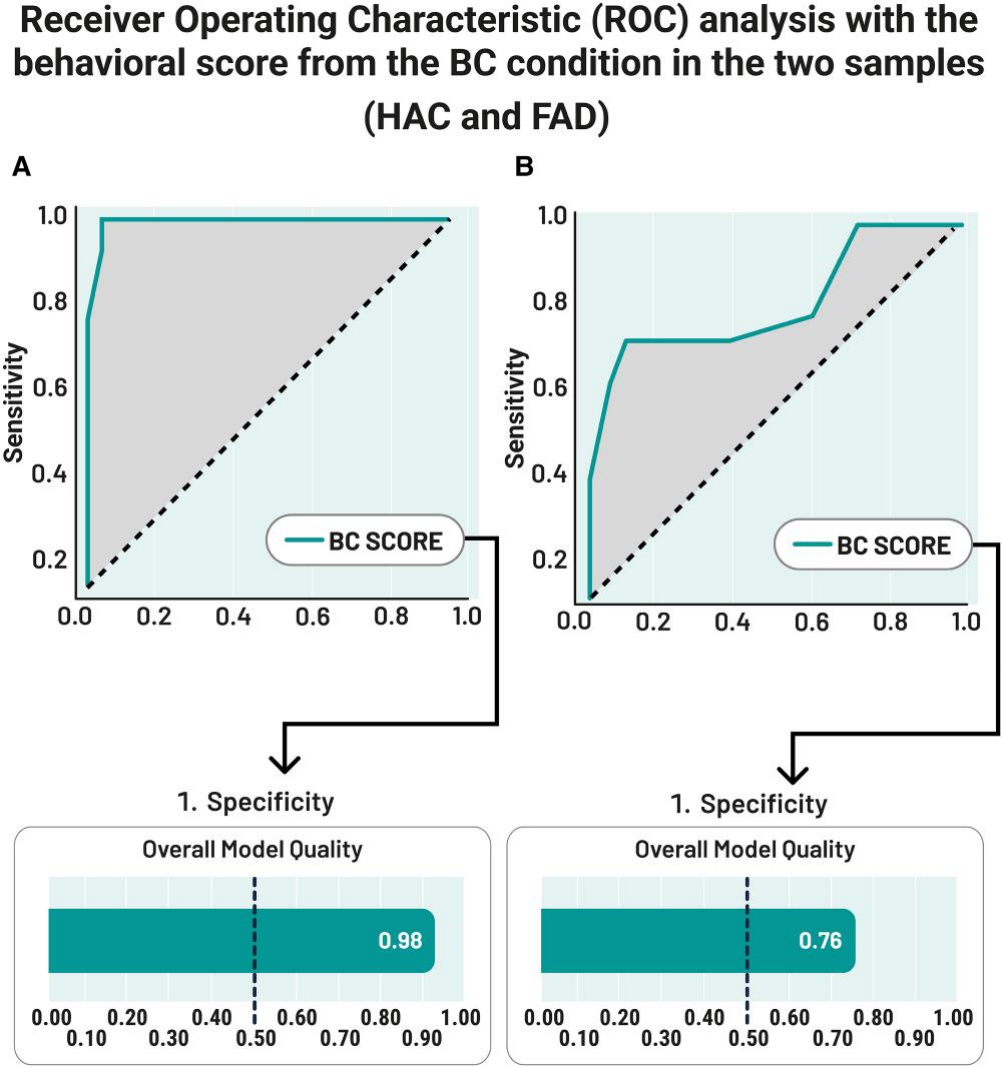
**Results from ROC analysis entering the BC score for (A) Controls FAD versus FAD (AUROC = 1) and (B) Controls HCA versus HCA (AUROC = 0.98).** Panels Overall Model Quality: A value <0.5 indicates the model is no better than random prediction.

The BC Score achieved an Area Under the Curve (AUC) of 99.4% for the classification of Controls FAD versus FAD (sensitivity = 100%, specificity = 93% and accuracy = 98%) and 82.3% for the classification of Controls HCA versus HCA (sensitivity = 79%, specificity = 67% and accuracy = 73%). Both models were well above chance (a good model has a value above 0.5; the Controls FAD versus FAD model achieved a 0.98 quality score, and that of Controls HCA versus HCA achieved a 0.76 quality score; see Fig. 3).

Binomial correlations with FDR corrections applied were conducted to explore potential associations between VSTM functions, as assessed via UC Score, the BC Score, the Cost of Binding, IQ Raven Matrices and Years of Education. IQ and Years of Education were significantly correlated (*r* = 0.42, *P* < 0.001). While both the UC and BC scores correlated with IQ (*r* = 0.51, *P* < 0.001 and *r* = 0.39, *P* < 0.001) and Years of Education (*r* = 0.45, *P* < 0.001 and *r* = 0.37, *P* < 0.001), denoting the influence of such factors on general VSTM abilities, the Cost of Binding did not (IQ: *r* = 0.06, *P* = 0.55; Years of Education: *r* = 0.02, *P* = 0.85) (see Supplementary Fig. 3 for the correlation graphs and results from whole-sample correlations). These results, along with the outcomes from the GLM, which controlled for the influence of years of education, confirm the insensitivity of VSTM binding functions to such factors.

### AI classification for eye-tracking data

**Figure 4:**
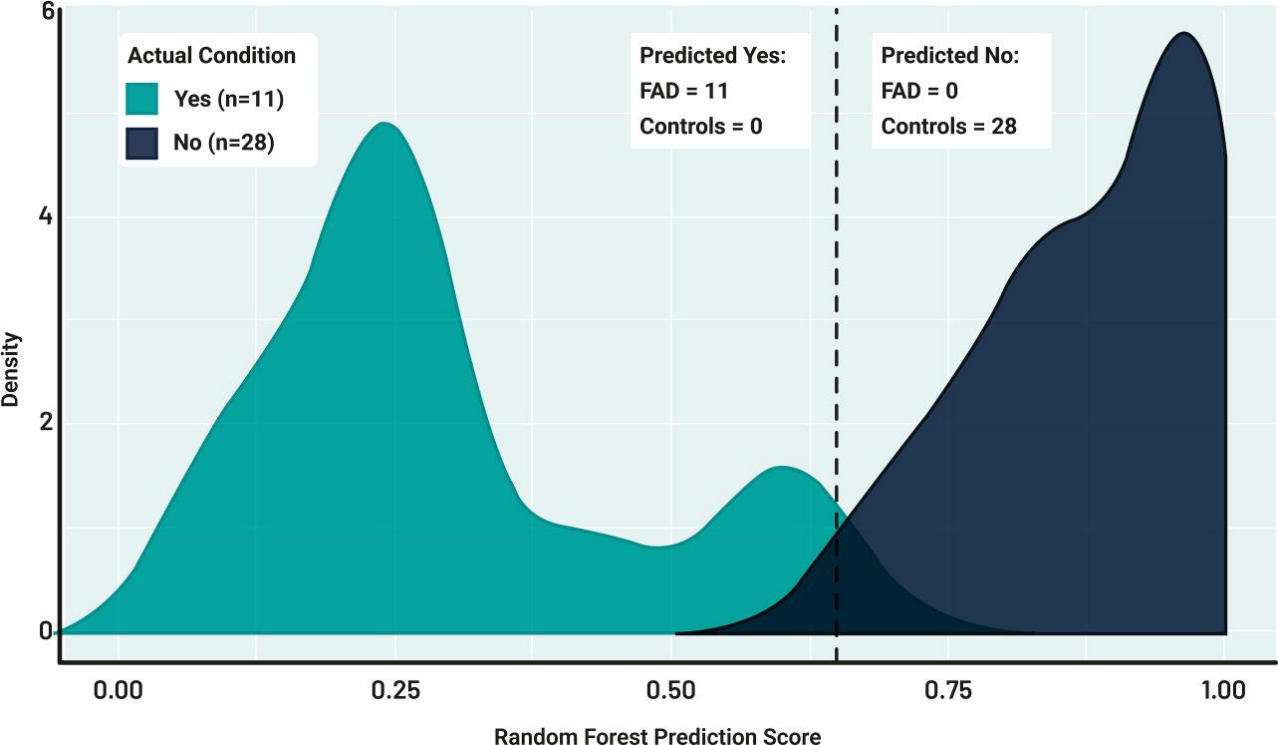
**RF model distribution when separating FAD from controls.** The model considers the output of the RF prediction. The dotted line corresponds to the calculated threshold (0.62).

We first explored whether the AI algorithm could accurately discriminate between FAD and their corresponding controls. For a threshold of 0.62, 28 controls and 11 FAD were correctly classified (see Fig. 4). FAD classification metrics were: TPR = 100%, FPR = 0%, TNR = 100%, FNR = 0%, NPV = 100%, PPV = 100%, cost = 0% (showing how inaccurate the model is in terms of its ability to estimate the relationship between the predicted value and the actual value) and accuracy = 100%. The RF threshold that yielded the above metrics was 0.62 (see Supplementary Fig. 4 for detailed classification outcomes).

**Figure 5:**
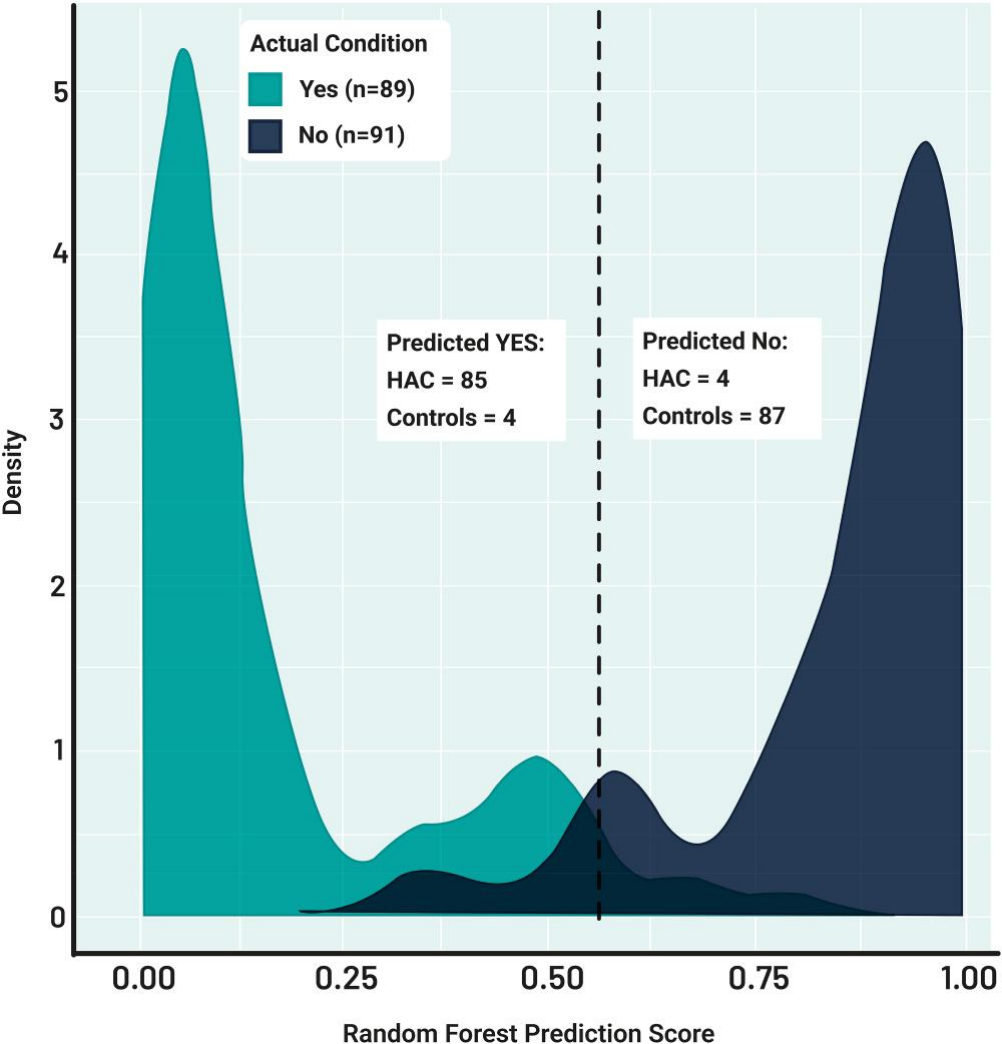
**RF model distribution when separating HAC from controls.** The model considers the output of the RF prediction. The dotted line corresponds to the calculated threshold (0.71).

When HAC and their corresponding controls entered the RF algorithm, the classification accuracy was 96%. For a threshold of 0.55, 87 out of 91 Controls and 85 out of 89 HAC were correctly classified (see Fig. 5). HAC classification metrics were: TPR = 94.5%, FPR = 3.37%, TNR = 96.6%, FNR = 5.49%, NPV = 94.5%, PPV = 96.6% and cost = 4.44% (see Fig. 4 and Supplementary Fig. 5 for detailed classification outcomes).

## Discussion

The present study was set out to investigate the hypothesis that the novel neurocognitive biomarker investigated here (i.e. VSTMB and ET powered by AI), would enhance the sensitivity of the assessment, and reveal impairments in asymptomatic carriers who are undetected by traditional neuropsychological and behavioural assessments. Our key findings were, first, we confirmed in this new study that VSTMB (i.e. BC) is a function affected by the E280A-PSEN1 FAD from its asymptomatic to its symptomatic stages. Second, AI RF classification confirmed that the new cognitive biomarker, which combines behavioural and ET measures, considerably outperforms pure behavioural outcomes. Classification metrics confirmed that 100% of FAD patients and 96% of HAC were correctly classified. Finally, the above findings were unaccounted for by education or CR. We discuss these findings in turn.

Our results reflect a substantial improvement in detection and classification achieved by the novel cognitive biomarker. Relative to the results reported by Parra *et al*.,^11^ our current results indicate a better classification accuracy for FAD patients even though the disease severity of our current patients (MMSE = 24.36 ± 3.64) and that of those seen by Parra *et al*.^11^ (MMSE = 25.55 ± 3.69) was similar [BC condition reported by Parra *et al*.^11^: FAD versus Controls for BC, AUC = 0.84 (*P* < 0.001); Asymptomatic Carriers versus Controls, AUC = 0.86 (*P* < 0.001)]. Traditional cognitive assessments for AD focus on memory, language, executive functions and visuospatial abilities.^46^ Although such assessments have proved valuable in detecting and monitoring dementia, they are insensitive to capture the early preclinical stages.^47^ Our understanding of cognition in general and of memory in particular has improved considerably since these traditional tests were first developed two or three decades ago. We are now in the position not only to detect memory impairments, but to isolate and measure the integrity of cognitive functions that operate on memory (i.e. binding) even when memory as a background ability remains unaffected. As we demonstrated in the present study, 100% of FAD and 96% of HAC performed below the neurocognitive cut-off value for binding (i.e. BC) despite these participants being in different predementia stages of the E280A-PSEN1 FAD variant.^23^ It is worth noting that all HAC were undetected by traditional neuropsychological and clinical assessment (see Supplementary Table 1), thus highlighting the combined sensitivity and specificity that this novel domain-specific neurocognitive marker is able to achieve (see Randolph^48^ for a discussion on domain-specific versus composite scores).

Traditional and emergent views emphasize the role of the hippocampus as a structure seemingly explaining the earliest cognitive and functional impairments in AD.^49^ Accrued evidence suggests that this notion has been misleading, as regions of the medial temporal lobe (MTL) other than the hippocampus, and from outside the MTL, are targeted by AD much earlier in its preclinical stages.^50^ The role of the eye as a biomarker for AD has been recently acknowledged,^51^ and we have previously reported that ET measures vastly outperform pure behavioural scores drawn from the VSTMB paradigm.^2^ In the context of the oculomotor behaviours investigated here, accumulating evidence suggests that deep cortico-subcortical interactions play a significant role. For example, some consider the locus coeruleus a prodromal AD biomarker.^52^ The locus coeruleus is a subcortical hub of the noradrenergic pathway known to be involved in pupil regulation and cognitive performance. Future research should address the interplay of these cortico-subcortical interactions as potential underpinnings of VSTMB functions and their consistent sensitivity to the very early stages of AD.

Not only has this combined approach (VSTMB + ET) accurately identified HAC who will inevitably develop FAD, but it has also prospectively predicted AD dementia in sporadic MCI patients who were followed up for 36 months, achieving a PPV of 94% and an NPV of 100%.^2^ In the neuroimaging field, atrophy in the perirhinal, entorhinal and basal forebrain regions has been suggested as a potential biomarker signature.^53^ Using AI, morphological abnormalities in the hippocampus, entorhinal cortex, basal ganglia, precuneus and the cerebellum have been identified in the pre-clinical stages of AD.^54^ Korolev *et al*.^55^ used more than 750 variables, including clinical, MRI and plasma biomarkers, to predict progression from MCI to AD dementia. They identified cognitive and functional parameters as the most predictive. This evidence is encouraging for two reasons. First, it suggests that the complex associations between cognitive functions and brain pathology underpinning the sensitivity of this novel neurocognitive biomarker to AD (e.g. cortico-subcortical interactions during VSTMB) can be potentially revealed via AI methods. Second, it suggests that the quest for domain-specific cognitive and functional abilities should continue.

Our RF models used behavioural and ET data from a single cognitive domain, that is, VSTMB. This is different from previous models, which combined either multidimensional data (i.e. clinical, neuroimaging and cognitive) or data from different cognitive assessments, aiming at achieving high sensitivity. By developing better intelligent algorithms that rely on and thoroughly exploit information from single neurocognitive domains, we can help to better understand the earliest manifestations of dementia, which can improve clinical care and assist with the development of new interventions. The AI-powered cognitive biomarker reported here achieved an FPR of 3.34% and an FNR of 5.49%. This level of precision is high and comparable with that of existing and emerging AD biomarkers. For example, Aβ-PET is a prohibitively expensive screening tool used in clinical trials. In the A4 trial,^6^ it reported an Aβ-PET screening fail rate of 71% with 3.39 individuals screened to identify one Aβ+ individual. Amyloid PET cost-effectiveness for diagnosis and clinical management^56^ remains extremely low. The advent of peripheral biomarkers (e.g. blood-based, ocular, such as OCT, etc.) calls for more integrative approaches that can help identify optimal, affordable and culture-free outcome measures that can contribute to effective screening tools to help prevent dementia.

An ongoing effort to revise the criteria for the appropriate use of amyloid PET^57^ revealed data that, for amyloid PET, the rates of positivity in late-onset MCI range from 45% to 70%. Between 15% and 20% of individuals clinically diagnosed with late-onset probable AD dementia have a negative amyloid PET. The levels of plasma Aβ42/Aβ40 are decreased by only 8–15% in individuals with cerebral amyloid pathology, compared to the 40–60% decreases seen in CSF. However, the % p-tau217 has detected both amyloid PET and tau PET positivity with an AUC above 95%. Mohs *et al*.^58^ found that the AUC for p-tau217 with amyloid positivity in various ethnic groups was above 80%. Blood-based biomarkers are now being considered to assess the effects of amyloid-β antibodies on the pathophysiology of AD in its preclinical stage.^57^ Nevertheless, we still face a gap regarding the availability of reliable neurocognitive markers for AD.

Extensive research carried out in this population of autosomal dominant AD has confirmed that the cognitive and clinical phenotypes of the AD continuum in the course of the E280A-PSEN1 mutation (familial) and sporadic AD are similar. Previous studies using the VSTMBT have confirmed this notion.^11,12^ Notably, using a different working memory binding task in carriers of various mutations linked to autosomal dominant AD, visuospatial binding deficits have also been reported by Liang *et al*.,^59^ confirming that such deficits reflect a cognitive phenotype of AD, regardless of its clinical variant and the task used for assessment. Considering recent developments in the field of peripheral biomarkers for AD (i.e. blood based and oculomotor), together with the need to include cognition in emergent diagnostic frameworks, we feel compelled to suggest that, by combining the AI-powered cognitive biomarker with other affordable biomarkers, such as those based on biofluids, the precision of preclinical detection protocols could be significantly enhanced in an accessible and representative manner.

This study has some limitations which are worth acknowledging. Our sample of FAD patients and their corresponding controls were small, but our large sample of HAC displayed a profile comparable to that seen in FAD cases. This, together with the classification accuracy achieved by our models, suggests that this pattern (VSTMB + ET) is typical of individual cases, not just reflecting group-level effects. We did not have data from other AD biomarkers available from the investigated sample. However, the presence of a single autosomal dominant mutation (E280A-PSEN1), which inevitably leads to FAD, provides reassurance that AD was the underlying condition affecting both FAD and HAC cases. The fact that we have achieved high and similar clinical classification powers across a largely unbalanced sample of FAD and HAC, being the latter group much larger, is of relevance to the field of AI-based prediction models for healthcare in general, and dementia prediction and prevention in particular. Finally, control participants recruited at AXIS Neuroscience underwent only cognitive screening and a clinical interview to determine normality (but see Supplementary Table 4 for data from controls recruited at the Neuroscience Centre of Antioquia). Although their scores on the MMSE fell within normal ranges, the test's limited sensitivity may have yielded false negatives. This seems unlikely, given the high classification accuracy of the RF models reported here. This is an approach we have reliably followed in the past.^11^ Nevertheless, future studies may consider administering the complete protocol to the entire sample to confirm the validity of our results.

## Conclusion

This is the first study that combines ET, VSTMB and AI algorithms to identify individuals who meet the criteria for HAC and FAD linked to the mutation E280A-PSEN1. The results presented here confirm that eye movement analysis powered by AI can outperform traditional forms of assessment in the detection of FAD before clinically meaningful symptoms emerge. This approach can drive research into the early detection of dementia, assist with more efficient resource utilization and also inform clinical practice with greater accuracy.

## Supplementary Material

fcaf370_Supplementary_Data

## Data Availability

Data from Grupo de Neurociencias, Facultad de Medicina, Universidad de Antioquia can be made available conditionally to data sharing agreements in accordance with data privacy statements signed by the patients within the legal framework of the General Data Protection Regulation of Colombia.
