# Supplementary material for: Eye movements powered by artificial intelligence identify asymptomatic carriers of familial Alzheimer’s disease
Source: Brain Commun. 2025 Sep 25;7(5):fcaf370. doi: 10.1093/braincomms/fcaf370 (PMC12528986; doi:10.1093/braincomms/fcaf370)
Supplement: fcaf370_Supplementary_Data [file fcaf370_supplementary_data.docx]

**Eye movements powered by artificial intelligence identify asymptomatic carriers of familial Alzheimer’s disease**

**Gerardo Fernández, Luis Mendez, Francisco Lopera, David Aguillon, and Mario A Parra**

**Supplementary Material**


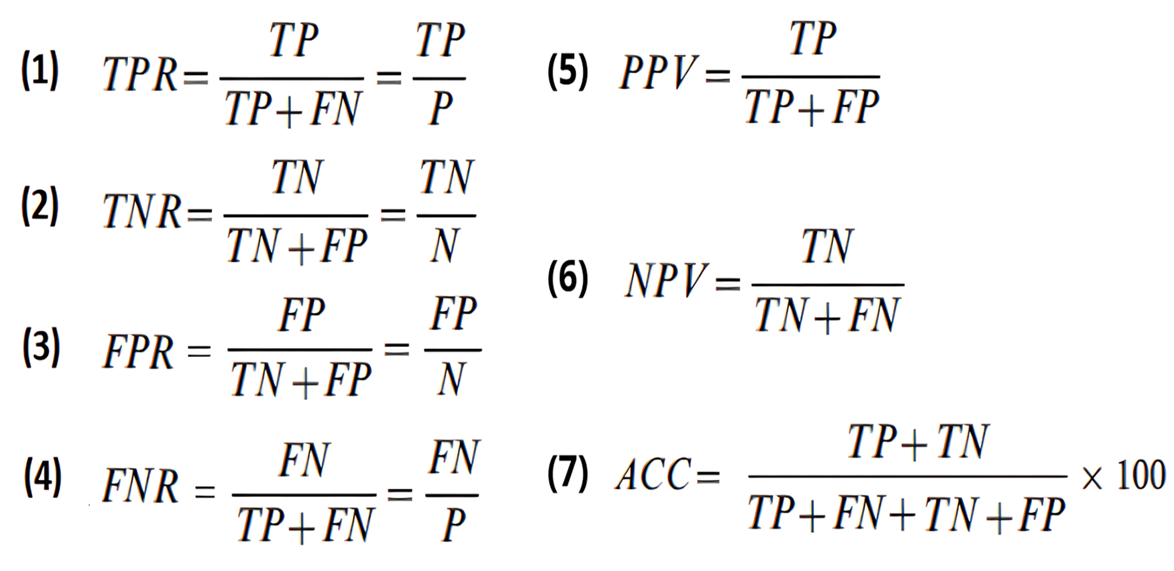


**Supplementary Figure 1.** Equations used for the calculation of classification performance of behavioral and RF outcomes. P is the number of positive cases in the sample, including true positives – TP – and False Negatives – FN. N is the number of negative cases in the sample, including true negatives – TN – and false positives – FP. TPR is the probability that an actual positive case will test positive, and TNR is the probability that an actual negative case will test negative. FPR is the rate at which negative cases are incorrectly classified as positive, and FNR is the rate at which positive cases are incorrectly classified as negative. ACC reflects the percentage of correctly classified cases as positive and negative among all positive (P) and all negative (N) cases.


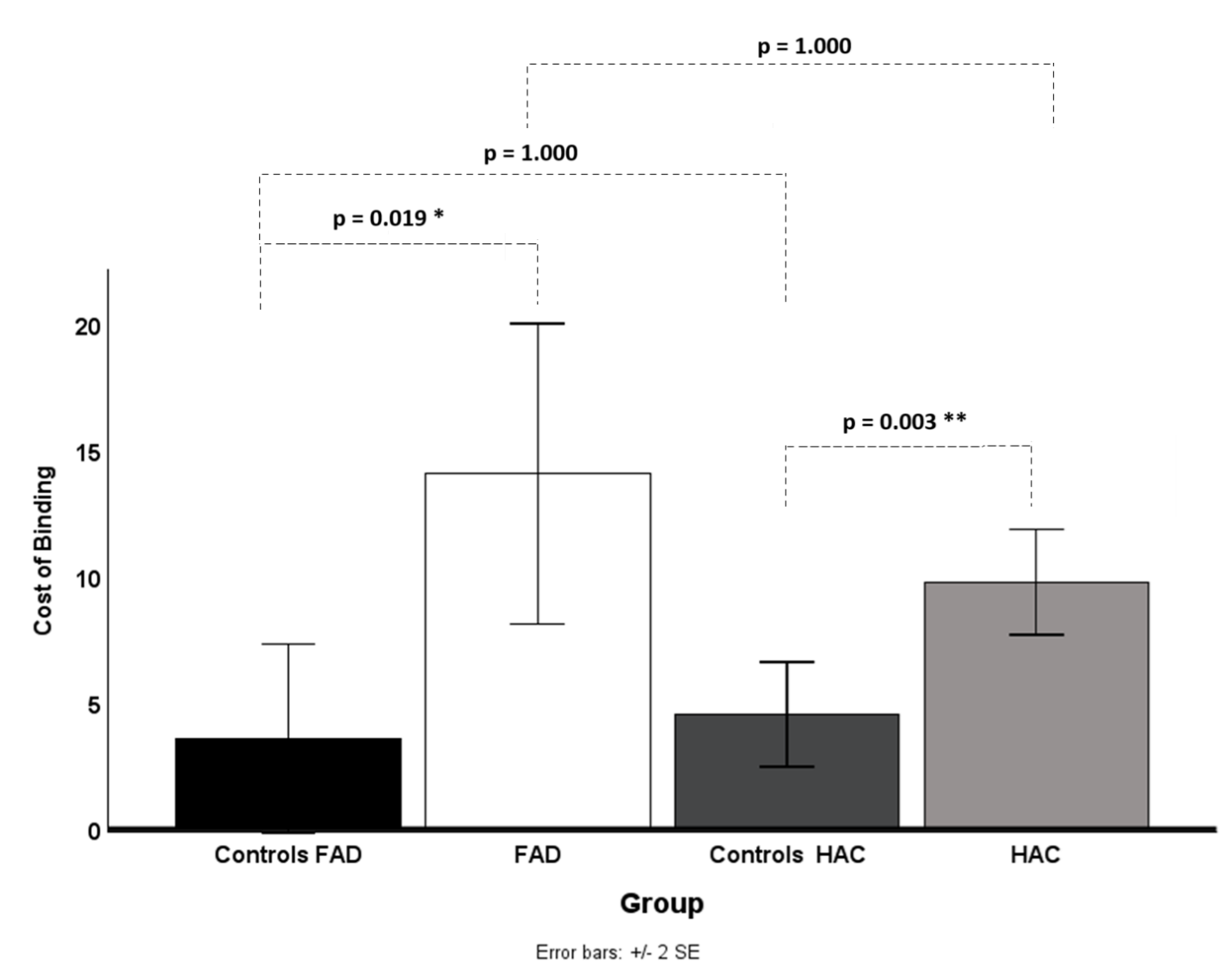


**Supplementary Figure 2**. Results from the GLM with Group (Controls FAD vs FAD vs Controls HCA vs HCA) as the between-subjects factor, years of education and age as covariates and the Cost of Binding as the dependent variable. The GLM revealed a significant effect of Group [*F*(3,215) = 4.99, p < 0.001 , η^2^ = 0.105, β = 0.98]. Bonferroni corrected pos-hoc tests confirmed Controls FAD vs FAD were significantly different (Mean Difference = 10.48, p = 0.019, CI= -19.83/-1.125). Controls HAC vs HAC were also significantly different (Mean Difference = 5.25, p = 0.003, CI= -9.17/-1.33). Neither the Control groups (Mean Difference = 0.94, p = 1.000, CI= -6.63/4.73) nor the Carrier Groups (Mean Difference = 4.28, p = 1.000, CI= -4.12/12.68) significantly differed. We, therefore, decided to subject the BC Score to ROC analysis.

**
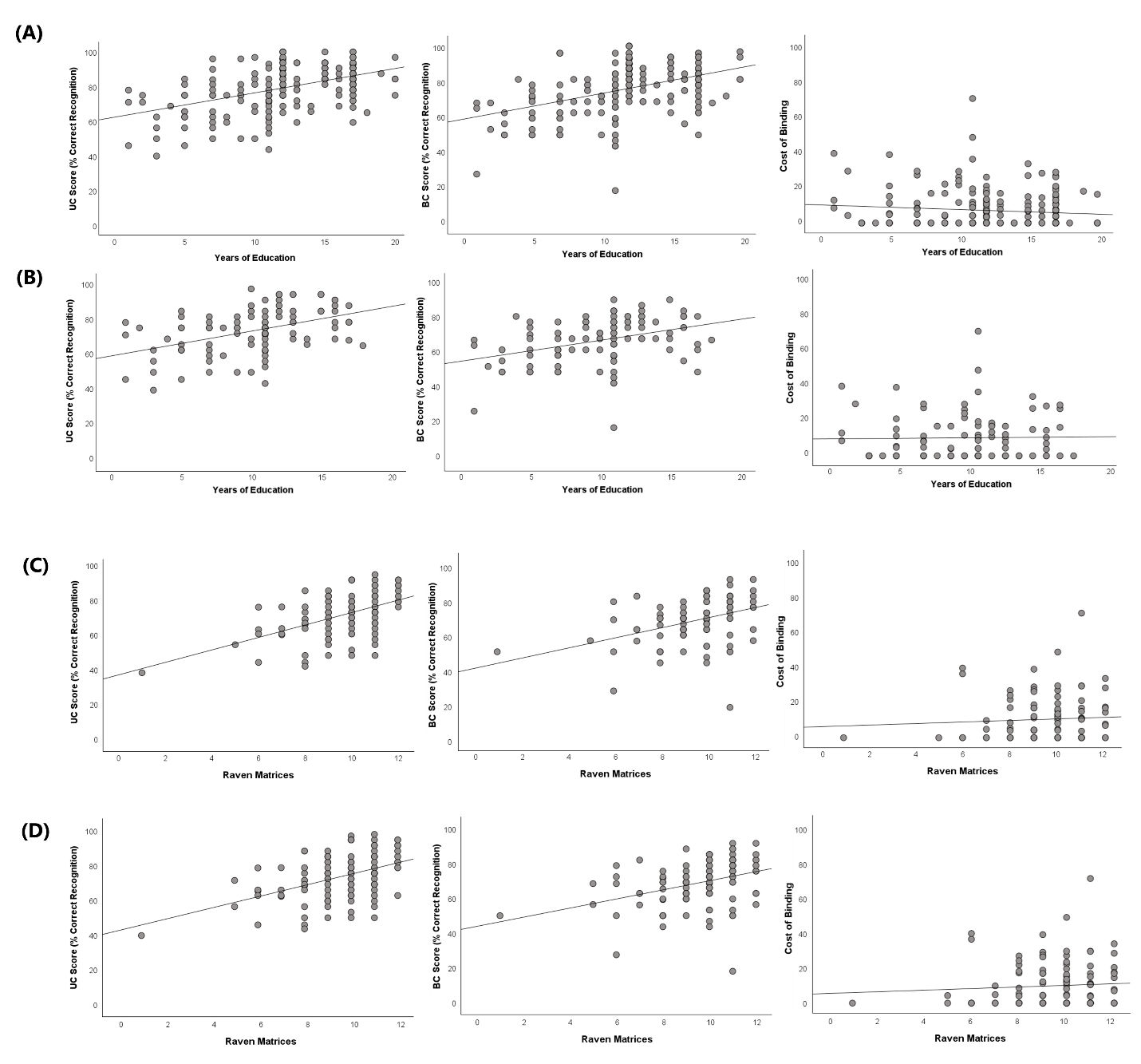
**

**Supplementary Figure 3**. Results from the Pearson bivariate correlation analyses between Years of Education, IQ measure Raven Matrices, and variables from the VSTMBT (% Correct Recognition on the UC and BC condition, and the Cost of Binding), including the whole sample (Panels A and C) and the mutation carrier samples (Panels B and D). Years of Education and IQ significantly correlated in the whole (*r*=0.42, *p*<0.001) and in the mutation carrier sample analysis (*r*=0.44, *p*<0.001). Correlations across samples (whole and mutation carriers) and Years of Education and IQ yielded similar results. Mutation carriers’ data showed that Years of Education and variables of the VSTMBT significantly correlated after FDR correction (UC: *r*=0.45, *q*<0.001; BC: *r*=0.37, *q*<0.001), except for the Cost of Binding (*r*=0.02, *q*=0.855). The same pattern was observed for the whole-sample analysis (UC: *r*=0.44, *q*<0.001; BC: *r*=0.46, *q*<0.001; Cost of Binding: *r*=-0.01, *q*=0.132). Correlations with mutation carriers’ data between the IQ measure and variables of the VSTMBT proved significant after FDR correction (UC: *r*=0.51, *q*<0.001) and (BC: *r*=0.39, *q*<0.001), except for the Cost of Binding (*r*=0.06, *q*=0.647). Again, the same pattern was observed for the whole-sample analysis (UC: *r*=0.46, *q*<0.001; BC: *r*=0.37, *q*<0.001; Cost of Binding: *r*=0.07, *q*=0.493).

**
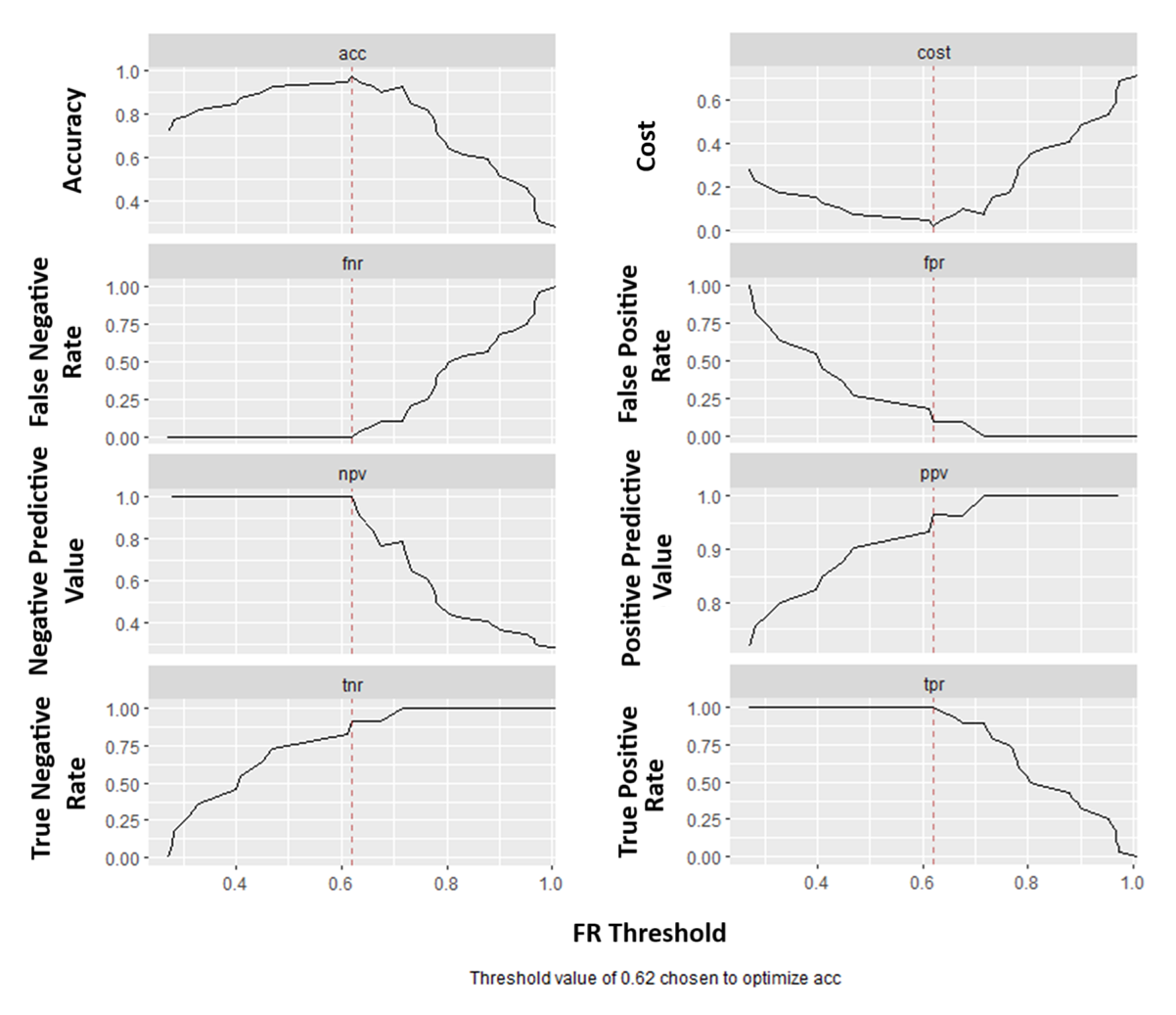
**

**Supplementary Figure 4.** Parameters used to evaluate the performance of the RF when classifying FAD from Controls (see caption of Supplementary Figure 1 for the formulas used to calculate the above classification parameters and their description. their values are presented in the text of the manuscript). Abbreviations: acc = Accuray; cost = Cost; tnr = True Negative Rate; fpr = False Positive Rate; npv = Negative Predictive Value; ppv = Positive Predictive Ratio; tnr = True Negative Rate; tpr = True Positive Rate. See Supplementary Table 4 for Model performance, including the AUC of the ROC analysis.

**
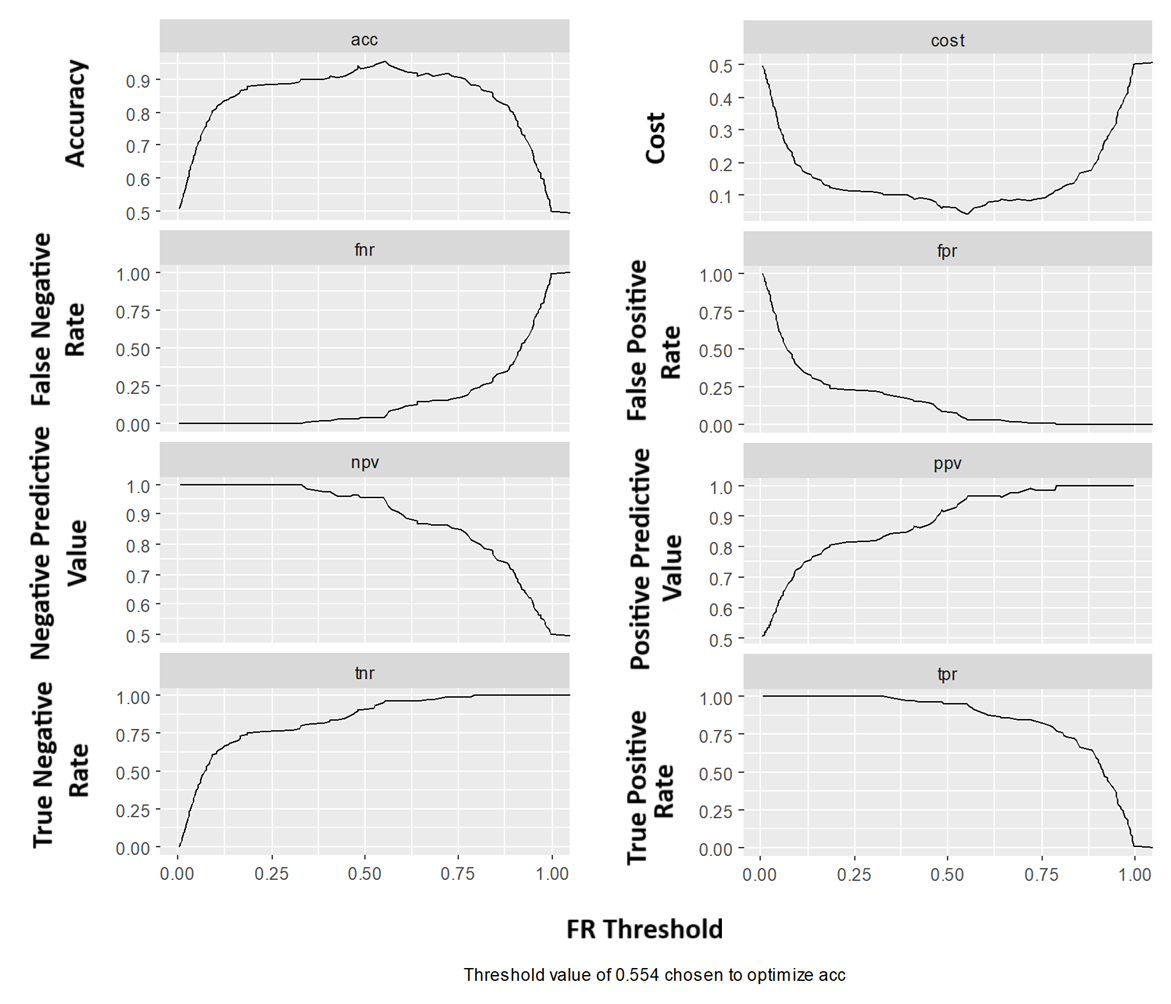
**

**Supplementary Figure 5**. Parameters used to evaluate the performance of the RF when classifying HAC from Controls (see caption of Supplementary Figure 1 for the formulas used to calculate the above classification parameters and their description. their values are presented in the text of the manuscript). Abbreviations: acc = Accuray; cost = Cost; tnr = True Negative Rate; fpr = False Positive Rate; npv = Negative Predictive Value; ppv = Positive Predictive Ratio; tnr = True Negative Rate; tpr = True Positive Rate. See Supplementary Table 3 for Model performance, including the AUC of the ROC analysis.

**Supplementary Table 1**. Clinical classification of mutation carriers based on the criteria proposed by (Acosta-Baena et al.^1^. AC: Asymptomatic Carrier; Asymp-preMCI: Asymptomatic pre-MCI; Symp-preMCI: Symptomatic pre-MCI; FAD: familial Alzheimer’s disease; MCI: Mild-cognitive impairment. The Asymp-preMCI, Symp-preMCI, and MCI entered our FAD Group. The AC entered the Healthy Carriers Group.

| **N** | **Clinically significant cognitive decline** | **Memory complaints** | **Memory complaints with effect** | **Impairment in complex instrumental functions** | **Impairment in basic activities of daily living** | **DSM-IV dementia criteria** | **Classification** |
| --- | --- | --- | --- | --- | --- | --- | --- |
| 1 | 1 | 1 | 1 | 1 | 1 | 1 | FAD |
| 2 | 1 | 1 | 1 | 1 | 1 | 1 | FAD |
| 3 | 1 | 1 | 1 | 1 | 1 | 1 | FAD |
| 4 | 1 | 1 | 1 | 1 | 1 | 1 | FAD |
| 5 | 1 | 1 | 1 | 0 | 0 | 0 | MCI |
| 6 | 1 | 0 | 1 | 0 | 0 | 0 | Symp-preMCI |
| 7 | 1 | 1 | 1 | 1 | 0 | 0 | MCI |
| 8 | 1 | 0 | 0 | 0 | 0 | 0 | Asymp-preMCI |
| 9 | 1 | 1 | 1 | 1 | 1 | 1 | FAD |
| 10 | 1 | 1 | 1 | 1 | 0 | 0 | MCI |
| 11 | 1 | 1 | 1 | 1 | 1 | 1 | FAD |
| 1 | 0 | 0 | 0 | 0 | 0 | 0 | AC |
| 2 | 0 | 0 | 0 | 0 | 0 | 0 | AC |
| 3 | 0 | 0 | 0 | 0 | 0 | 0 | AC |
| 4 | 0 | 0 | 0 | 0 | 0 | 0 | AC |
| 5 | 0 | 0 | 0 | 0 | 0 | 0 | AC |
| 6 | 0 | 0 | 0 | 0 | 0 | 0 | AC |
| 7 | 0 | 0 | 0 | 0 | 0 | 0 | AC |
| 8 | 0 | 0 | 0 | 0 | 0 | 0 | AC |
| 9 | 0 | 0 | 0 | 0 | 0 | 0 | AC |
| 10 | 0 | 0 | 0 | 0 | 0 | 0 | AC |
| 11 | 0 | 0 | 0 | 0 | 0 | 0 | AC |
| 12 | 0 | 0 | 0 | 0 | 0 | 0 | AC |
| 13 | 0 | 0 | 0 | 0 | 0 | 0 | AC |
| 14 | 0 | 0 | 0 | 0 | 0 | 0 | AC |
| 15 | 0 | 0 | 0 | 0 | 0 | 0 | AC |
| 16 | 0 | 0 | 0 | 0 | 0 | 0 | AC |
| 17 | 0 | 0 | 0 | 0 | 0 | 0 | AC |
| 18 | 0 | 0 | 0 | 0 | 0 | 0 | AC |
| 19 | 0 | 0 | 0 | 0 | 0 | 0 | AC |
| 20 | 0 | 0 | 0 | 0 | 0 | 0 | AC |
| 21 | 0 | 0 | 0 | 0 | 0 | 0 | AC |
| 22 | 0 | 0 | 0 | 0 | 0 | 0 | AC |
| 23 | 0 | 0 | 0 | 0 | 0 | 0 | AC |
| 24 | 0 | 0 | 0 | 0 | 0 | 0 | AC |
| 25 | 0 | 0 | 0 | 0 | 0 | 0 | AC |
| 26 | 0 | 0 | 0 | 0 | 0 | 0 | AC |
| 27 | 0 | 0 | 0 | 0 | 0 | 0 | AC |
| 28 | 0 | 0 | 0 | 0 | 0 | 0 | AC |
| 29 | 0 | 0 | 0 | 0 | 0 | 0 | AC |
| 30 | 0 | 0 | 0 | 0 | 0 | 0 | AC |
| 31 | 0 | 0 | 0 | 0 | 0 | 0 | AC |
| 32 | 0 | 0 | 0 | 0 | 0 | 0 | AC |
| 33 | 0 | 0 | 0 | 0 | 0 | 0 | AC |
| 34 | 0 | 0 | 0 | 0 | 0 | 0 | AC |
| 35 | 0 | 0 | 0 | 0 | 0 | 0 | AC |
| 36 | 0 | 0 | 0 | 0 | 0 | 0 | AC |
| 37 | 0 | 0 | 0 | 0 | 0 | 0 | AC |
| 38 | 0 | 0 | 0 | 0 | 0 | 0 | AC |
| 39 | 0 | 0 | 0 | 0 | 0 | 0 | AC |
| 40 | 0 | 0 | 0 | 0 | 0 | 0 | AC |
| 41 | 0 | 0 | 0 | 0 | 0 | 0 | AC |
| 42 | 0 | 0 | 0 | 0 | 0 | 0 | AC |
| 43 | 1 | 0 | 0 | 0 | 0 | 0 | Asymp-preMCI |
| 44 | 0 | 0 | 0 | 0 | 0 | 0 | AC |
| 45 | 0 | 0 | 0 | 0 | 0 | 0 | AC |
| 46 | 0 | 0 | 0 | 0 | 0 | 0 | AC |
| 47 | 0 | 0 | 0 | 0 | 0 | 0 | AC |
| 48 | 0 | 0 | 0 | 0 | 0 | 0 | AC |
| 49 | 0 | 0 | 0 | 0 | 0 | 0 | AC |
| 50 | 0 | 0 | 0 | 0 | 0 | 0 | AC |
| 51 | 0 | 0 | 0 | 0 | 0 | 0 | AC |
| 52 | 0 | 0 | 0 | 0 | 0 | 0 | AC |
| 53 | 0 | 0 | 0 | 0 | 0 | 0 | AC |
| 54 | 0 | 0 | 0 | 0 | 0 | 0 | AC |
| 55 | 0 | 0 | 0 | 0 | 0 | 0 | AC |
| 56 | 1 | 0 | 0 | 0 | 0 | 0 | Asymp-preMCI |
| 57 | 0 | 0 | 0 | 0 | 0 | 0 | AC |
| 58 | 0 | 0 | 0 | 0 | 0 | 0 | AC |
| 59 | 0 | 0 | 0 | 0 | 0 | 0 | AC |
| 60 | 0 | 0 | 0 | 0 | 0 | 0 | AC |
| 61 | 0 | 0 | 0 | 0 | 0 | 0 | AC |
| 62 | 0 | 0 | 0 | 0 | 0 | 0 | AC |
| 63 | 0 | 0 | 0 | 0 | 0 | 0 | AC |
| 64 | 0 | 0 | 0 | 0 | 0 | 0 | AC |
| 65 | 0 | 0 | 0 | 0 | 0 | 0 | AC |
| 66 | 0 | 0 | 0 | 0 | 0 | 0 | AC |
| 67 | 1 | 0 | 0 | 0 | 0 | 0 | Asymp-preMCI |
| 68 | 0 | 0 | 0 | 0 | 0 | 0 | AC |
| 69 | 0 | 0 | 0 | 0 | 0 | 0 | AC |
| 70 | 0 | 0 | 0 | 0 | 0 | 0 | AC |
| 71 | 0 | 0 | 0 | 0 | 0 | 0 | AC |
| 72 | 0 | 0 | 0 | 0 | 0 | 0 | AC |
| 73 | 0 | 0 | 0 | 0 | 0 | 0 | AC |
| 74 | 0 | 0 | 0 | 0 | 0 | 0 | AC |
| 75 | 0 | 0 | 0 | 0 | 0 | 0 | AC |
| 76 | 0 | 0 | 0 | 0 | 0 | 0 | AC |
| 77 | 0 | 0 | 0 | 0 | 0 | 0 | AC |
| 78 | 0 | 0 | 0 | 0 | 0 | 0 | AC |
| 79 | 0 | 0 | 0 | 0 | 0 | 0 | AC |
| 80 | 0 | 0 | 0 | 0 | 0 | 0 | AC |
| 81 | 0 | 0 | 0 | 0 | 0 | 0 | AC |
| 82 | 0 | 0 | 0 | 0 | 0 | 0 | AC |
| 83 | 1 | 0 | 0 | 0 | 0 | 0 | Asymp-preMCI |
| 84 | 0 | 0 | 0 | 0 | 0 | 0 | AC |
| 85 | 0 | 0 | 0 | 0 | 0 | 0 | AC |
| 86 | 0 | 0 | 0 | 0 | 0 | 0 | AC |
| 87 | 0 | 0 | 0 | 0 | 0 | 0 | AC |
| 88 | 0 | 0 | 0 | 0 | 0 | 0 | AC |
| 89 | 0 | 0 | 0 | 0 | 0 | 0 | AC |

**Supplementary Table 2.** Data collected with the two ET, GazePoint and the Head-Mounted Display (HMD) Hewlett-Packard, in control participants.

|  | **GazePoint (n = 55)** | | **HP (n = 36)** | | **Stat** | | |  |
| --- | --- | --- | --- | --- | --- | --- | --- | --- |
|  | **Mean** | **SD** | **Mean** | **SD** | | ***t*** | **p-value** | |
| Fixation Duration | 389.08 | 95.74 | 348.05 | 49.88 | | 1.81 | 0.060 | |
| Number of Fixations | 0.22 | 0.06 | 0.20 | 0.04 | | 1.33 | 0.180 | |
| Pupil | 0.01 | 0.01 | 0.01 | 0.01 | | 0.12 | 0.910 | |
| Gazing | 18.38 | 25.23 | 11.65 | 4.92 | | 1.26 | 0.210 | |

We analyzed whether the two Eye Trackers contributed comparable data in a selected sample of healthy control participants. None of the variables used in the study showed significant differences. Except for a few variables that heavily rely on the sampling frequency, most ET metrics are becoming increasingly compatible across ET devices, including handheld devices ^2,3^.

**Supplementary Table 3.** Out-of-fold performance of all trained models when classifying HAC from Controls**.** Models tuned via 5-fold cross validation over 9 combinations of hyperparameter values. Best performance: AUPR = 0.93, AUROC = 0.98. By Random Forest with hypermarameters: mtry = 4, splitrule = extratrees, min.node.size = 1.

| Mtry | splitrule | min.node.size | AUROC | Sens | Spec | ROCSD | SensSD | SpecSD |
| --- | --- | --- | --- | --- | --- | --- | --- | --- |
| 4 | extratrees | 1 | 0.984 | 0.956 | 0.922 | 0.0192 | 0.0609 | 0.0493 |
| 2 | extratrees | 10 | 0.98 | 0.944 | 0.865 | 0.0216 | 0.0962 | 0.0294 |
| 4 | gini | 2 | 0.977 | 0.922 | 0.922 | 0.0268 | 0.093 | 0.115 |
| 3 | gini | 11 | 0.976 | 0.922 | 0.911 | 0.0293 | 0.0633 | 0.0843 |
| 1 | gini | 8 | 0.976 | 0.933 | 0.899 | 0.0278 | 0.0913 | 0.0457 |
| 1 | gini | 7 | 0.976 | 0.933 | 0.899 | 0.0265 | 0.0913 | 0.0457 |
| 1 | extratrees | 10 | 0.961 | 0.933 | 0.865 | 0.0268 | 0.0994 | 0.0294 |
| 1 | extratrees | 17 | 0.959 | 0.922 | 0.876 | 0.0256 | 0.093 | 0.0243 |
| 1 | extratrees | 18 | 0.958 | 0.922 | 0.854 | 0.0287 | 0.093 | 0.0487 |

**Supplementary Table 4.** Out-of-fold performance of all trained models when classifying FAD from Controls**.** Models tuned via 5-fold cross validation over 9 combinations of hyperparameter values. Best performance: AUPR = 0.82, AUROC = 1. By Random Forest with hypermarameters: mtry = 1, splitrule = extratrees, min.node.size = 2.

| mtry | splitrule | min.node.size | AUROC | Sens | Spec | ROCSD | SensSD | SpecSD |
| --- | --- | --- | --- | --- | --- | --- | --- | --- |
| 7 | extratrees | 9 | 1 | 1 | 0.933 | 0 | 0 | 0.149 |
| 6 | gini | 1 | 1 | 1 | 1 | 0 | 0 | 0 |
| 5 | gini | 1 | 1 | 1 | 1 | 0 | 0 | 0 |
| 4 | gini | 11 | 1 | 1 | 1 | 0 | 0 | 0 |
| 1 | gini | 20 | 1 | 1 | 0.733 | 0 | 0 | 0.2527 |
| 1 | gini | 13 | 1 | 1 | 0.833 | 0 | 0 | 0.2357 |
| 1 | gini | 10 | 1 | 1 | 0.833 | 0 | 0 | 0.2357 |
| 1 | extratrees | 2 | 1 | 1 | 0.833 | 0 | 0 | 0.2357 |
| 1 | extratrees | 12 | 0.986 | 1 | 0.733 | 0.0298 | 0 | 0.2527 |

**Supplementary Table 5.** Neuropsychological assessment of Control participants recruited in the Neuroscience Centre of Antioquia.

|  | **Controls FAD (n=2)** | | | **Controls HAC (n=11)** | | |
| --- | --- | --- | --- | --- | --- | --- |
|  | **Mean** | **SD** | **# below norms** | **Mean** | **SD** | **# below norms** |
| MMSE | 29.71 | 0.81 | 1.00* | 29.62 | 0.68 | 0.00 |
| World List Learning (Total) (CERAD) | 14.50 | 0.71 | 0.00 | 21.64 | 4.25 | 0.00 |
| ROF Copy | 27.00 | 1.41 | 0.00 | 31.14 | 4.70 | 0.00 |
| ROF Recall | 17.00 | 2.83 | 0.00 | 20.18 | 6.81 | 0.00 |
| TMTA (time) | 121.50 | 81.32 | 1.00* | 44.55 | 17.90 | 0.00 |
| FAS | 26.00 | 2.83 | 0.00 | 36.91 | 10.41 | 0.00 |
| Category Fluency (CERAD) | 15.50 | 4.95 | 0.00 | 21.45 | 4.46 | 0.00 |
| WCST Categories | 3.00 | 0.00 | 1.00* | 3.91 | 1.97 | 0.00 |
| WCST Perseverations | 22.00 | 8.49 | 0.00 | 13.36 | 6.04 | 0.00 |
| WAISIII Digit to Symbol | 28.00 | 12.73 | 0.00 | 56.55 | 15.12 | 0.00 |
| Raven Matrices | 7.50 | 3.54 | 1.00* | 9.82 | 1.89 | 0.00 |
| IADL | 7.50 | 0.71 | 0.00 | 8.00 | 0.00 | 0.00 |
| QMF | 16.50 | 12.02 | 1.00* | 3.38 | 6.05 | 0.00 |
| QMP | 15.50 | 13.44 | 0.00 | 8.82 | 5.21 | 0.00 |

* One participant showed borderline performance and was attributed to low levels of education (2 years).

**REFERENCES**

1. Acosta-Baena, N., Sepulveda-Falla, D., Lopera-Gomez, C. M., Jaramillo-Elorza, M. C., Moreno, S., Aguirre-Acevedo, D. C.,…Lopera, F. (2011). Pre-dementia clinical stages in presenilin 1 E280A familial early-onset Alzheimer's disease: a retrospective cohort study. Lancet Neurol, 10(3), 213-220. https://doi.org/S1474-4422(10)70323-9 [pii];10.1016/S1474-4422(10)70323-9 [doi]
2. Stein, N., Niehorster, D. C., Watson, T., Steinicke, F., Rifai, K., Wahl, S., & Lappe, M. (2021). A Comparison of Eye Tracking Latencies Among Several Commercial Head-Mounted Displays. I-Perception, 12(1). https://doi.org/10.1177/2041669520983338
3. Titz J, Scholz A, Sedlmeier P. (2018s). Comparing eye trackers by correlating their eye-metric data. Behav Res Methods, 50(5):1853-1863. https://doi.org/10.3758/s13428-017-0954-y
